# Better survival after transcatheter aortic valve replacement by process improvements

**DOI:** 10.1007/s12471-020-01526-7

**Published:** 2020-12-07

**Authors:** G. J. van Steenbergen, D. van Veghel, D. N. Schulz, M. Soliman-Hamad, P. A. Tonino, S. Houterman, L. Dekker, NHR THI Registration Committee

**Affiliations:** 1 Department of Cardiology, Catharina Hospital, Eindhoven, The Netherlands; 2 Department of Cardiothoracic Surgery, Catharina Hospital, Eindhoven, The Netherlands; 3 Netherlands Heart Registration (NHR), Utrecht, The Netherlands; 4 Department of Biomedical Technology, Eindhoven University of Technology, Eindhoven, The Netherlands

**Keywords:** Cardiology, TAVR, Quality improvement, Outcome-based quality improvement, Value based health care, Quality committee, Multidisciplinary meeting

## Abstract

**Objective:**

The aim of this study is to assess the effects on procedural, 30-day, and 1‑year all-cause mortality by a newly introduced quality improvement strategy in patients after transcatheter aortic valve replacement (TAVR).

**Methods:**

In October 2015, a coherent set of quality improving interventions with respect to patient geriatric screening, general diagnostic examination and safety of the procedure was implemented at a single centre in the Netherlands. Patients undergoing TAVR in 2013–2018 were included for retrospective analysis. Mortality was assessed in the pre-quality improvement strategy cohort (January 2013 to October 2015; cohort A) and in the post-quality improvement strategy cohort (November 2015 to December 2018; cohort B). Logistic regression analysis was used to estimate the influence of patient and procedural characteristics on the results of the quality improvement strategy in terms of procedural, 30-day, and 1‑year all-cause mortality.

**Results:**

In total, 806 patients were analysed with 274 patients in cohort A and 532 patients in cohort B. After introduction of the quality improvement strategy, procedural (4.4% to 1.3%, *p* < 0.01), 30-day (8.4% to 2.7%, *p* < 0.01) and 1‑year (16.4% to 8.5%, *p* < 0.01) all-cause mortality significantly decreased. Multivariate regression analysis showed that the quality improvement strategy also significantly reduced 30-day (odds ratio [OR] 0.19, 95% confidence interval [CI] 0.09–0.42) and 1‑year (OR 0.38, 95% CI 0.24–0.61) all-cause mortality if corrected for patient characteristics.

**Conclusion:**

Structural meetings on evaluation of outcomes highlight potential areas for improvement and subsequent outcome-based quality improvement initiatives can result in lower procedural, 30-day, and 1‑year all-cause mortality.

**Electronic supplementary material:**

The online version of this article (10.1007/s12471-020-01526-7) contains supplementary material, which is available to authorized users.

## Whats new?

- Embedded structural outcome monitoring in TAVR patients provides insight in areas for improvement
- An outcome-based quality improvement strategy can result in lower procedural, 30-day, and 1‑year all-cause mortality in TAVR patients
- There is a positive temporal trend in the procedural, 30-day, and 1‑year all-cause mortality of TAVR patients in the Netherlands

## Introduction

Transcatheter aortic valve replacement (TAVR) is a relatively new, yet recommended alternative for conventional aortic valve surgery (SAVR) in high-risk patients [1, 2]. After the European introduction of TAVR in 2007, utilisation of this therapy rapidly expanded and a vast body of evidence is now available that depicts a trend in improving clinical outcomes after TAVR [3, 4]. Nevertheless, some post-procedural adverse events remain prevalent, such as atrioventricular block and stroke, and costs remain high [5].

In 2006, Porter and Teisberg introduced value-based health care (VBHC) as a strategy to solve the emerging cost crisis in health care without compromise in quality of provided care. In VBHC, the overarching goal for all involved parties is to put patient value, defined as outcomes that matter most to patients divided by costs of health-care delivery, central in health care [6]. To aid benchmarking outcomes in TAVR patients, standardised outcomes have been defined for use in clinical trials and national cardiac registries, such as the Netherlands Heart Registration (NHR). The NHR facilitates value-based outcome monitoring for cardiac interventions [7]. The use of its uniform data is considered essential for systematically monitoring outcomes relevant to patients and has previously shown to be a valuable tool for achieving insight in patient outcomes [8–10]. Nevertheless, structural meetings to discuss outcomes are not yet embedded in all cardiac centres in the Netherlands [11].

In the current retrospective study, we aim to evaluate a real-world, outcome-based quality improvement strategy in a single centre and we will illustrate that this quality improvement strategy reduces procedural, 30-day, and 1‑year all-cause mortality after TAVR. To assess the accuracy of our findings, we contrast our regional results with the national TAVR registry.

## Methods

This study is conducted at the Catharina Heart Centre in Eindhoven, the Netherlands. At our centre, using value-based health-care principles, we evaluate local real-world outcome data in comparison with the national benchmark provided by the Netherlands Heart Registration (NHR) every two weeks in a multidisciplinary quality committee. The committee is assigned to initiate quality assessment projects and evaluations to ensure continuous improvement of outcomes that matter most to patients.

### Quality improvement strategy

In 2015, the quality committee identified the mortality rate after TAVR as an improvement topic. Based on expert opinion and available evidence at the time, the quality committee drafted an improvement strategy with the aim to reduce all-cause mortality in TAVR patients. Consequently, this strategy was assessed by management staff for feasibility.

Consensus was reached to implement each of the following strategies in daily practice in October 2015:

- Pre-procedural patient assessment in a dedicated TAVR outpatient clinic for all patients [12];
- Geriatric screening based on age (>80 years) or high frailty score: items from the comprehensive geriatric assessment [13] have been associated with mortality and major adverse cardiac events (MACE) after the procedure [14, 15] and geriatricians are advised to be involved in heart team decisions concerning aortic valve disease management [16];
- Complete valve sizing and catheter access evaluation based on a computed tomography (CT) scan for all patients: CT provides an accurate measurement of the annulus and detailed information about peripheral access [17, 18];
- Two operators instead of one in case of high risk or complex cases;
- Implementation of TAVR procedures without general anaesthesia if possible: local anaesthesia and conscious sedation have been associated with shorter hospital stay, shorter procedural time and decreased mortality [12, 19, 20];
- Monthly multidisciplinary evaluation of procedural and clinical TAVR pathway: implemented in compliance with increased awareness of quality assessment in health care.

During the study period, the workflow for TAVR implantation was otherwise not structurally altered.

### Inclusion criteria

For this study, we included all patients undergoing TAVR in accordance with the Heart Team decision at our centre from 2013 to 2018. Two cohorts are distinguished: the pre-quality improvement strategy cohort with TAVR between January 2013 and October 2015 (cohort A) and the post-quality improvement strategy cohort with TAVR between November 2015 and December 2018 (cohort B).

### Definitions and outcomes

Mortality is defined as all-cause mortality during follow-up confirmed by the Personal Records Database (BRP) from the Dutch government [21]. Procedural mortality encompasses all-cause mortality during the procedure and in the first 3 days thereafter. To assess cardiac surgical risk, the logistic European System for Cardiac Operative Risk Evaluation I (EuroSCORE I) as proposed by Roques et al. is used (EuroSCORE II is not available for cohort A) [21, 22]. Left ventricular ejection fraction (LVEF) is determined no earlier than 6 months prior to TAVR and determined by echocardiography or, if available, cardiac magnetic resonance imaging. The most recently reported pre-procedural value is used for this study. Urgent procedures are non-elective and are required during the current admission of the patient [21]. Emergency procedures cannot wait until the following day after the decision to perform the procedure is made [21]. We used the third universal definition of myocardial infarction by Thygesen et al. [21, 23]. Stroke is defined as permanent neurological dysfunction, diagnosed by a consultant neurologist, as a result of cerebral, spine or retinal damage caused by acute infarction [21, 24]. A distinction was made between self-expandable valves (Evolut R, Medtronic Inc., Minneapolis, MN, USA), balloon-expendable valves (Sapian 3, Edwards Lifesciences, Irvine, CA, USA) and valves used for study purposes (Direct Flow Medical, Inc., Santa Rosa, CA, USA). Other clinical parameters are defined by the NHR and are aligned with the valve academic research consortium (VARC) and International Consortium for Health Outcomes Measurement (ICHOM) and, therefore, reflect international standards for outcome monitoring in TAVR patients [21, 25–28].

### Statistical analysis

Patient characteristics are expressed as mean values with corresponding standard deviation (SD) for continuous normal distributed data, median with interquartile range (IQR) for continuous non-normal distributed data and as absolute and relative frequencies for categorical data. Chi-squared test, Mann-Whitney U test or Student’s t‑test were used to make a comparison between the two cohorts, where appropriate. A cumulative sum control chart (CUSUM) is used to display the trend in mortality over the period of interest. In the CUSUM curve, mortality is compared with a predetermined reference. If the mortality rate is lower than predicted, the curve increases with the predicted change of death. The curve decreases with *1‑change of death* if the mortality rate is higher. Every TAVR patient between January 2013 and December 2018 is chronologically presented and each case either results in an upslope (survival) or downslope (death). The mean mortality rate at our centre up to implementation of the quality improvement strategy was used as a reference.

Using univariate logistic regression analysis, the relationship between all-cause mortality and the quality improvement strategy was explored. Subsequent bivariate logistic regression analysis was performed with mortality as dependent variable and the quality improvement strategy and patient characteristics as independent variables. Variables with a *p*-value ≤0.1 in bivariate analysis were considered for inclusion in the multivariate model besides external clinical judgment. A *p*-value <0.05 was considered significant.

To assess relevance of decrease in mortality at our centre, the national secular trend in mortality rates is calculated for procedural, 30-day and 1‑year all-cause mortality. This national trend is represented by 11 other heart centres in the Netherlands performing TAVR in the period 2013–2018 (data for 2018 are not available for 1‑year mortality analysis) and originates from the NHR [21]. For each individual patient, the predicted mortality is estimated, using logistic regression analysis and includes the following patient characteristics: chronic pulmonary disease, previous cardiac surgery, previous stroke, sex, age, LVEF, creatinine clearance and year of intervention. For this study, stratification was performed based on the period in which TAVR was performed in concurrence with cohort A and cohort B. Further sub-stratification between our centre and the other centres combined was performed to compare local with national results. The mean predicted mortality (with standard deviations) and crude mortality data for each cohort are depicted in a histogram for visual comparison with standard errors and standard deviations respectively. Pearson’s chi-squared test was used to assess the differences between crude all-cause mortality rates across local and national cohorts. All analyses were performed using SPSS 25 (SPSS Inc., Chicago, Illinois, USA).

## Results

### Patient characteristics

In total, 806 patients met the inclusion criteria (Tab. 1). Cohort A consisted of 274 patients and cohort B included 532 patients. In cohort B, 192 patients who underwent TAVR in 2018 had not completed 1‑year follow-up at the time of writing. Patients in cohort A more frequently had prior stroke (13.3% vs 7.5%; *p* = 0.011) and more often lower NYHA class (61.8% vs 52.8% for class I–II and 38.2% vs 47.2% for class III–IV; *p* = 0.023) compared with patients in cohort B. Patients in cohort A more frequently had LVEF <30% (8.8% vs 7.3%, *p* < 0.01). In cohort B more patients had a logistic EuroSCORE I > 10 (75.9% vs 68.2%, *p* = 0.018) and significantly more patients underwent urgent procedures compared with cohort A (16.6% vs 7.7%; *p* < 0.01). There was a significant difference between the two cohorts regarding the implanted valve type which favours self-expandable valves in cohort A (60.2% vs. 44.7% in cohort B; *p* < 0.01).

**Table 1:** Patient, procedural, and outcome characteristics stratified by pre- and post-quality improvement strategy

Variable^a^	Cohort A *N* = 274	Cohort B *N* = 532	*P*-value
Male	133 (48.7)	293 (55.0)	0.12
Age, years	80.1 ± 6.3	80.8 ± 6.1	0.14
BMI, kg/m^2^	26.9 ± 4.6	27.0 ± 4.4	0.56
*NYHA class*			0.023
I–II	162 (61.8)	280 (52.8)	
III–IV	100 (38.2)	250 (47.2)	
LVEF, %	55 (14–70)	55 (19–84)	0.53
<30%	24 (8.8)	37 (7.3)	<0.01
Logistic EuroSCORE I	17.9 ± 13.4	17.9 ± 11.6	0.21
>10	187 (68.2)	404 (75.9)	0.018
Creatinine, µmol/l	107.7 ± 57.4	109.3 ± 61.7	0.33
Dialysis	3 (1.1)	4 (0.8)	0.24
Diabetes mellitus	72 (26.6)	134 (25.3)	0.73
Chronic pulmonary disease	51 (18.8)	93 (17.5)	0.70
Peripheral arterial disease	72 (26.6)	171 (32.3)	0.089
Previous stroke	36 (13.3)	40 (7.5)	0.011
Previous cardiac surgery	67 (24.6)	154 (29.1)	0.18
Previous aortic valve surgery	20 (7.5)	29 (5.5)	0.28
Myocardial infarction lasts 90 days	2 (0.7)	15 (2.8)	0.068
*Urgency of procedure*			<0.01
Elective	251 (92.3)	435 (82.2)	
Urgent	21 (7.7)	88 (16.6)	
Emergent	0 (0.0)	6 (1.1)	
Type of implanted valve			<0.01
Self-expandable	165 (60.2)	238 (44.7)	
Balloon-expandable	85 (31.0)	293 (55.1)	
Direct flow medical	24 (8.8)	1 (0.2)	
*Mortality*			
Procedural	12 (4.4)	7 (1.3)	<0.01
30-day	23 (8.4)	14 (2.7)	<0.01
1‑year	45 (16.4)	45 (8.5)^b^	<0.01

*BMI* body mass index, *NYHA* New York Heart Association, *LVEF* left ventricular injection fraction, *EuroSCORE* European System for Cardiac Operative Risk Evaluation, *SD* standard deviation, *IQR* interquartile range

^a^ Data are presented as mean ± SD, median (IQR) or *n* (%)

^b^ *N* = 340

### Outcome

**Fig. 1:**
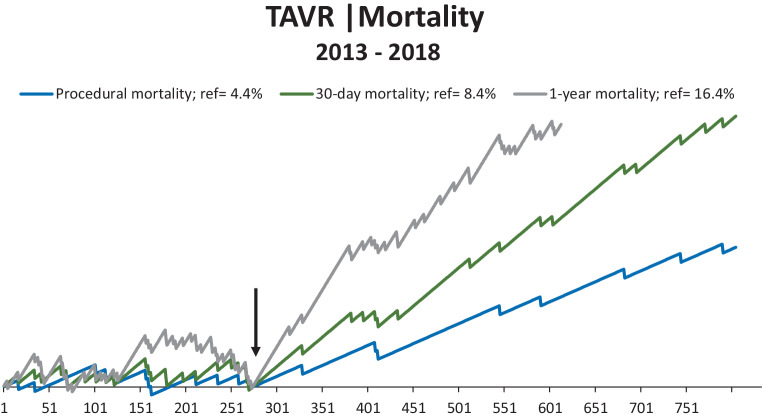
CUSUM chart of procedural, 30-day and 1‑year all-cause mortality. ↓ = date of implementation of quality improvement strategy

The CUSUM curve of procedural, 30-day and 1‑year all-cause mortality is depicted in Fig. 1. A gradual improvement after the implementation of the quality improvement strategy (arrow) is shown. In comparison with cohort A, procedural, 30-day, and 1‑year all-cause mortality decreased significantly in cohort B: 4.4% to 1.3% (*p* < 0.01), 8.4% to 2.7% (*p* < 0.01) and 16.4% to 8.5% (*p* < 0.01) respectively (Tab. 1).

In univariate logistic regression analysis, the quality improvement strategy is associated with procedural (odds ratio [OR] 0.29, 95% confidence interval [CI] 0.11–0.75), 30-day (OR 0.29, 95% CI 0.15–0.58) and 1‑year all-cause mortality (OR 0.47, 95% CI 0.31–0.74) (Tab. 2).

**Table 2:** Bivariate analysis of procedural, 30-day, and 1‑year all-cause mortality

	Procedural			30-day			1‑year		
	OR	95% CI	*P-*value	OR	95% CI	*P-*value	OR	95% CI	*P-*value
Quality improvement strategy	0.29	(0.11–0.75)	0.011^a^	0.29	(0.15–0.58)	<0.01^a^	0.47	(0.31–0.74)	<0.01^a^
Age (years)	1.05	(0.97–1.14)	0.22	1.05	(0.99–1.12)	0.100	1.01	(0.98–1.05)	0.532
Male sex	0.94	(0.38–2.35)	0.89	0.79	(0.40–1.54)	0.484	0.58	(0.37–0.92)	0.021
LVEF <30%	NA			0.32	(0.04–2.39)	0.266	0.83	(0.34–1.99)	0.668
Creatinine (µmol/l)	0.99	(0.98–1.01)	0.37	0.99	(0.99–1.01)	0.575	1.00	(0.99–1.00)	0.265
BMI (kg/m^2^)	0.92	(0.81–1.05)	0.20	0.91	(0.84–0.99)	0.045	0.96	(0.91–1.02)	0.174
Diabetes mellitus	1.37	(0.51–3.68)	0.53	1.45	(0.71–2.95)	0.312	0.87	(0.52–1.47)	0.606
NYHA III–IV	1.28	(0.46–3.62)	0.63	1.56	(0.77–3.17)	0.219	1.02	(0.65–1.61)	0.936
Chronic pulmonary disease	1.49	(0.47–4.75)	0.49	1.65	(0.75–3.67)	0.214	2.00	(1.20–3.34)	<0.01
Previous stroke	NA			0.46	(0.11–1.99)	0.302	0.59	(0.25–1.42)	0.239
Urgent vs elective	2.18	(0.59–8.09)	0.24	2.89	(1.22–6.82)	0.016	2.33	(1.31–4.13)	<0.01
Balloon vs self-expandable valve	1.78	(0.68–4.70)	0.24	1.25	(0.63–2.05)	0.524	0.81	(0.51–1.29)	0.371

*OR* odds ratio, *CI* confidence interval, *LVEF* left ventricular ejection fraction, *BMI* body mass index, *NYHA* New York Heart Association, *NA* not applicable

^a^ Results of univariate analysis

In a bivariate model with the quality improvement strategy, BMI (OR 0.91, CI 0.84–0.99) and urgency (OR 2.89, 95% CI 1.22–6.82) were significantly associated with 30-day mortality (Tab. 2). Multivariate analysis showed that the quality improvement strategy (OR 0.19, 95% CI 0.09–0.42) was significantly associated with 30-day mortality if corrected for urgency of the procedure, age and BMI (Tab. 3).

**Table 3:** Multivariate regression analysis of 30-day and 1‑year all-cause mortality

	30-day			1‑year		
	OR	95% CI	*P*-value	OR	95% CI	*P*-value
Quality improvement strategy	0.19	(0.09–0.42)	<0.01	0.38	(0.24–0.61)	<0.01
Age, years	1.04	(0.98–1.11)	0.157			
Male sex				0.59	(0.37–0.96)	0.032
BMI, kg/m^2^	0.92	(0.84–1.01)	0.078			
Chronic pulmonary disease				3.29	(1.16–3.29)	0.011
Urgent vs elective	2.92	(1.22–6.97)	0.016	2.33	(1.30–4.17)	<0.01

*OR* odds ratio, *CI* confidence interval, *BMI* body mass index

Male sex, chronic pulmonary disease and urgency was significantly associated with 1‑year mortality in a bivariate model with the quality improvement strategy (Tab. 2). The quality improvement strategy was also significantly associated with 1‑year all-cause mortality when corrected for male sex, urgency of the procedure and chronic pulmonary disease in a multivariate model (OR 0.38, 95% CI 0.24–0.61; Tab. 3).

**Fig. 2:**
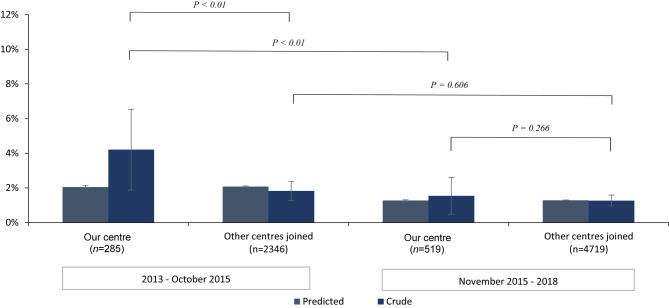
Comparison of procedural all-cause mortality at our centre and at the other centres combined

**Fig. 3:**
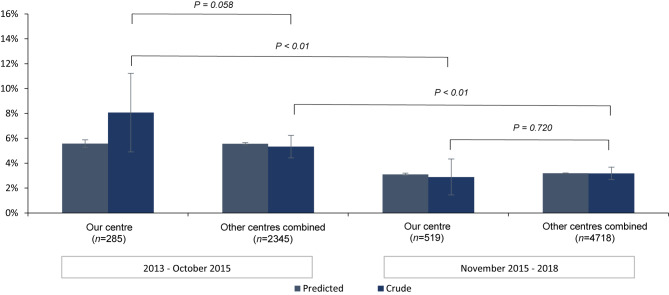
Comparison of 30-day all-cause mortality at our centre and at the other centres combined

**Fig. 4:**
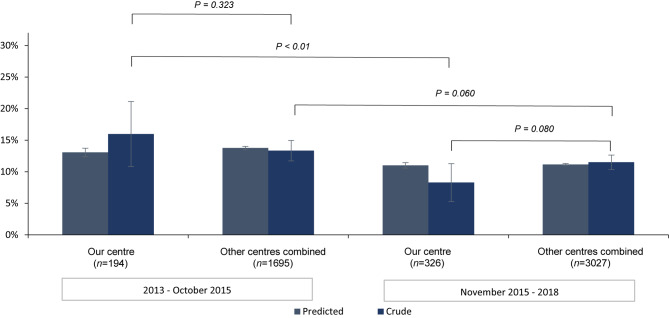
Comparison of 1‑year all-cause mortality at our centre and at the other centres combined

The crude procedural all-cause mortality in our centre compared with the other centres combined was not significantly different in the November 2015–2018 interval (*p* = 0.266), but higher within the 2013–October 2015 interval (*p* < 0.01; Fig. 2). For both intervals, the crude 30-day and 1‑year all-cause mortality was not significantly different between our centre and the other centres combined. Overlapping standard error bars also indicate a not significant difference between adjusted all-cause mortality at our centre and the other centres combined for both intervals (Figs. 3 and 4).

## Discussion

We highlight that routine collection of real-world data, including local and national outcomes, provides insight into areas for improvement. Our study underlines that outcome monitoring should not be a situational priority (e.g. for research purposes) but requires structural embedment in health-care organisations. The latter enabled us to timely implement a coherent set of improvement interventions and show the significant contribution of the improvement strategy to the reduction in procedural, 30-day, and 1‑year all-cause mortality. The strength of our study lies within the implementation of a coherent set of evidence-based interventions. In addition, due to our outcome-based quality improvement strategy, local mortality rates in our centre reduced more than the national average.

Even though the coherent nature of our improvement strategy proved fruitful in reduction of procedural, 30-day, and 1‑year all-cause mortality, it does not allow for estimation of the weight of each individual quality improvement intervention on the presented results. Nevertheless, the individually implemented interventions have been discussed previously and have shown to individually contribute to the improving trend of all-cause mortality in TAVR patients [3, 19, 20]. The effect of the improvement strategy is presumably attributed to the multidimensional aspect of the different interventions and their conjoined effect on all-cause mortality. Routine outcome evaluation, collaboration with geriatricians, routinely performed computed tomography prior to the procedure, local anaesthesia during the procedure and two operators instead of one during complex procedures all contributed to TAVR outcome improvement over the years.

Despite the significant association between the quality improvement strategy and lower all-cause mortality, the learning curve characteristics for TAVR procedures cannot be underestimated. Decreased procedural safety and higher mortality rates have been described in low volume centres (<50 procedures annually) [29, 30]. Outcomes between intermediate-volume and high-volume centres, on the other hand, do not differ [29]. We expect minimal interference of a learning curve effect on the present results because our centre evolved from an intermediate-volume centre in 2013 to a high-volume centre towards 2018 and only dedicated cardiologists performed TAVR procedures throughout the entire study period. In addition, one could argue that with the introduction of an improvement strategy with a focus on patient screening and selection from October 2015 onward might have introduced a preference of treating patients with less comorbidity. Both regression analysis and the observation that patients in cohort B more frequently had a EuroSCORE >10 advocate against selection of ‘lower’ risk patients after October 2015. Moreover, local procedural mortality rate declined significantly compared with the national average in this time frame, indicating the incremental value of our strategy in comparison with spontaneous trends.

The mortality rates we described are in corroboration with previously published data by registries (other than the NHR). In the period prior to 2015, 30-day mortality was 8.4%, comparable with the Belgian (10.1%) and slightly lower than the French national registry (12.7% in similar periods) [31, 32]. In a meta-analysis of 137 studies with over 90,000 TAVR patients, 30-day mortality was 2.27%, comparable with our last study period [4]. In the same group of patients 1‑year mortality after TAVR was 11.35%, which is slightly higher compared with our study in a similar time frame (8.5%) [4].

### Limitations

We performed a retrospective analysis of real-world data which is associated with shortcomings with respect to the level of evidence. Besides, technical improvements to the valves and deployment devices presumably contributed to the reduction in procedural mortality. Detailed data on valve edition (e.g. Sapian XT vs Sapian 3) or delivery device are lacking and are therefore not incorporated in this study. Lastly, in the near future, the IMPULSE trial will provide more insight on the added value of quality improvement of patient care in aortic valve stenosis patients on a large scale compared with our single centre intervention [33].

## Conclusion

We demonstrated that embedded structural outcome monitoring provides insight in areas for improvement. Furthermore, we demonstrated that an outcome-based quality improvement strategy can result in lower procedural, 30-day, and 1‑year all-cause mortality in TAVR patients. It is also applicable to other fields in medicine.

## Caption Electronic Supplementary Material

NHR THI Registration Committee members
