# Supplementary material for: Better survival after transcatheter aortic valve replacement by process improvements
Source: Neth Heart J. 2020 Dec 7;29(4):193–200. doi: 10.1007/s12471-020-01526-7 (PMC7991044; doi:10.1007/s12471-020-01526-7)
Supplement: Supplementary file 1 — NHR THI Registration Committee members [file 12471_2020_1526_MOESM1_ESM.docx]

**SUPPLEMENTARY MATERIAL**

NHR THI Registration Committee members

| Amsterdam UMC | M.M. | Vis |
| --- | --- | --- |
| MUMC+ | S. | Kats |
| Amphia | P. | den Heijer |
| Catharina Ziekenhuis | W.A.L. | Tonino |
| Erasmus MC | P.P.T. | de Jaegere |
| HagaZiekenhuis | C.E. | Schotborgh |
| Isala | V. | Roolvink |
| Leids Universitair Medisch Centrum | F. | van der Kley |
| Medisch Centrum Leeuwarden | F. | Porta |
| Medisch Spectrum Twente | M.G. | Stoel |
| OLVG | G. | Amoroso |
| St. Antonius Ziekenhuis | L. | Timmers |
| Radboudumc | H.R. | Gehlmann |
| UMC Utrecht | P.R. | Stella |
| Universitair Medisch Centrum Groningen | H.W. | van der Werf |
| Amsterdam UMC | J.S. | Lemkes |
